# Predictive algorithm for the regional spread of coronavirus infection across the Russian Federation

**DOI:** 10.1186/s12911-023-02135-1

**Published:** 2023-03-14

**Authors:** Andrey Reshetnikov, Vitalii Berdutin, Alexander Zaporozhtsev, Sergey Romanov, Olga Abaeva, Nadezhda Prisyazhnaya, Nadezhda Vyatkina

**Affiliations:** 1 Institute of Social Sciences, Sechenov First Moscow State Medical University, Moscow, Russian Federation; 2 Contract Department, Federal Budgetary Institution of Healthcare “Volga District Medical Center of the Federal Medical and Biological Agency”, Nizhny Novgorod, Russian Federation; 3 Department of Theoretical and Applied Mechanics, Federal State Budgetary Educational Institution of Higher Education “Nizhny Novgorod State Technical University Named After R.E. Alekseev”, Nizhny Novgorod, Russian Federation; 4 Department of Sociology of Medicine, Health Economics, and Health Insurance, Sechenov First Moscow State Medical University, Moscow, Russian Federation

**Keywords:** COVID-19, Dynamics of viral disease, Epidemiological model, Predictive algorithm

## Abstract

**Background:**

Outbreaks of infectious diseases are a complex phenomenon with many interacting factors. Regional health authorities need prognostic modeling of the epidemic process.

**Methods:**

For these purposes, various mathematical algorithms can be used, which are a useful tool for studying the infections spread dynamics. Epidemiological models act as evaluation and prognosis models. The authors outlined the experience of developing a short-term predictive algorithm for the spread of the COVID-19 in the region of the Russian Federation based on the SIR model: Susceptible (vulnerable), Infected (infected), Recovered (recovered). The article describes in detail the methodology of a short-term predictive algorithm, including an assessment of the possibility of building a predictive model and the mathematical aspects of creating such forecast algorithms.

**Results:**

Findings show that the predicted results (the mean square of the relative error of the number of infected and those who had recovered) were in agreement with the real-life situation: σ(I) = 0.0129 and σ(R) = 0.0058, respectively.

**Conclusions:**

The present study shows that despite a large number of sophisticated modifications, each of which finds its scope, it is advisable to use a simple SIR model to quickly predict the spread of coronavirus infection. Its lower accuracy is fully compensated by the adaptive calibration of parameters based on monitoring the current situation with updating indicators in real-time.

## Introduction

Infectious disease outbreaks are a complex phenomenon involving numerous interaction factors. According to virology researchers, the SARS-CoV-2 coronavirus, which causes the COVID-19 disease, has spread rapidly because it combines genomes with different properties from other coronavirus strains [1]. Regardless of the state of the global health infrastructure, the novel coronavirus disease poses a threat to all countries. Globally, the number of COVID-19 patients is rising rapidly [2].

Over 5.7 million new cases were recorded from July 4 to July 10, 2022, which is an increase of 6% from the week before. Over 9800 fatalities were reported, which was about the same as the previous week in terms of new fatalities. Globally, there had been over 6.3 million reported fatalities and slightly under 553 million confirmed cases as of July 10, 2022. The new COVID-19 coronavirus has proven to be highly contagious, though not the deadliest disease that the world has seen in decades. COVID-19 has a relatively long incubation period of 2 days to 2 weeks (5–7 days on average), during which carriers are already infected even without obvious symptoms. Fever, fatigue, breathing problems, and a dry cough are the main signs and symptoms of COVID-19. The breathing issue is more severe and intense than the others. There is also a lack of necessary devices to deal with the increased number of patients [3].

The world's healthcare systems were unprepared for the latest coronavirus outbreak. The majority of government efforts at the moment are focused on stopping the coronavirus's spread and identifying potential hot spots. Due to their required close proximity to probable coronavirus patients, healthcare workers and vital personnel are the most susceptible to coronavirus infections. Public healthcare services are unable to handle the rising patient load. Due to the excessive number of patients, the hospitals have soon become overcrowded, but there aren't enough ventilators, PPE, oxygen providers, etc. Some patients must lay on mattresses on the floor because the hospitals can't handle the surge of people. Due to the ongoing influx of new patients who are coronavirus-infected, hospitals have emerged as significant coronavirus carriers. The hospital administrative staff, surgeons, and health care providers have the highest risk of contracting an infection [4].

During the World Health Summit on October 25–27th, 2020, the WHO Director-General and a number of senior executives and experts urged all governments to invest more resources in innovation, research and solutions to combat COVID-19 [5]. However, the health systems of the vast majority of countries worldwide have been struggling to contain the spread of coronavirus. For example, the State of Israel has repeatedly imposed strict restrictive measures, up to the complete closure of the country's airports [6]. After coming to power in January 2021, the new U.S. administration also rushed to apply stricter restraint policies [7].

On July 20, 2022, 221,955 cases of COVID-19 infection were recorded in China. The total number of deaths from coronavirus infection was 5,213 people. There are 5,661 people in the active phase of the disease, of which 383 are in critical condition. The lethality rate is 2.35%. There is tension in the country that the infamous situation in Wuhan could resurface repeatedly after the easing of quarantine restrictions. The situation in China is closely monitored by other countries, as they may end up in an even worse situation in a few days. The actions taken in China may seem excessive, but the Chinese authorities attempt to avoid past miscalculations. In the early stages of the coronavirus outbreak in Hubei province, the magnitude of the issue was not adequately assessed, and countermeasures were thus delayed. An equally acute problem is observed in South Korea, Singapore, Taiwan, and Hong Kong, which so far have managed to contain the spread of coronavirus [8].

In Russia, a rapid spread of infection in 2020 has significantly changed the lifestyle of all social groups [9, 10] and forced to reorganize the health care system in all regions of the country. Despite the relatively stable situation, 18,504,729 confirmed cases had been reported in the country as of 20 July 2022. For all time, 381,997 people died, 17,919,843 recovered.

Judging by the epidemiological situation with coronavirus in Europe and the United States, as of July 2022, an increase in the incidence is also expected in the Russian Federation by the autumn–winter period. If transport links between countries had remained at the pre-pandemic level, then the increase in the incidence would have begun somewhat earlier [11]. After a large wave of the Omicron strain passed in the country at the beginning of 2022, collective immunity grew quite strongly in the population, which gradually begins to weaken. Russian virologists note that the Omicron variant is less dangerous for humans than the parallel strains Alpha and Delta. With Omicron, the mortality rate is 0.3–0.4%. At the beginning of the pandemic, when the original strain from Wuhan (China) was spreading, the mortality rate was 5–6%. However, around 800,000 people around the world continue to be infected with the coronavirus every day. Despite the more recent mild course of the disease with the Omicron strain, about 900 thousand people died in the world in the first half of 2022.

The Centaur subspecies of the Omicron strain can be deadlier than other variants. In addition, it is more contagious than its predecessors BA.1-BA.5, it can successfully bypass the formed immunity, therefore its spread in Russia causes serious concern. It is possible that in the coming months a new, more pathogenic line will appear. The further situation with the spread of coronavirus will depend on which line of evolution the virus will follow. It is almost impossible to control it. According to official statistics as of 07/19/2022 in Russia, 18,499,044 cases of COVID-19 infection were laboratory confirmed, of which 17,915,526 patients fully recovered, 381,960 deaths were recorded. In the period from July 18 to July 19, 2022, there were 4200 COVID-19 cases, 4186 recoveries, and 44 deaths (Table 1).

**Table 1:** Selective up-to-date data on the regions of the Russian Federation as of July 19, 2022

Region	Infected	Active cases	Died	Recovered	Mortality (%)
Russian Federation	18,499,044 + 4200	201,558–30	381,960 + 44	17,915,526 + 4186	2.06
Moscow	2,789,777 + 790	150,294–215	44,340 + 11	2,595,143 + 994	1.59
St. Petersburg	1,542,397 + 521	4,363 + 86	34,256 + 5	1,503,778 + 430	2.22
Moscow region	986,440 + 384	6046–9	15,182 + 1	965,212 + 392	1.54
Sverdlovsk region	449,704 + 169	1895 + 76	10,766 + 2	437,043 + 91	2.39
Nizhny Novgorod Region	425,237 + 16	992–50	11,589	412,656 + 66	2.73
Voronezh region	390,658 + 114	1275–42	8353	381,030 + 156	2.14
Samara Region	386,888 + 81	879 + 41	7918 + 2	378,091 + 38	2.05

Pandemics have been more frequent during the past few decades as a result of increased urbanization and international travel. It's critical to create models to predict the risk of an infectious disease spreading further since a disease that spreads in one area has the potential to become a pandemic with global humanitarian and economic consequences.

The study of population epidemics has a long history dating back to the work of Kermack in 1927, in terms of mathematics, outbreak modeling, and control. In the field of statistical physics of disordered systems, spreading phenomena have also received substantial study. In this context, of high relevance is predictive modeling of a new coronavirus infection outbreak at the level of a given region. A variety of mathematical algorithms can be used as tools to study the dynamics of the spread of infectious diseases since epidemiological models are used to predict and evaluate other pathogenic behaviors [12]. It is quite clear that the specifics of the COVID-19 pandemic process should be studied and analyzed thoroughly, involving the methodology of mathematical modeling. Its essence is to replace the original object with its abstract image by a more detailed examination of the model based on computer and logic algorithms and study the true outbreak process. This approach of design and prediction combines the benefits of theoretical constructions with experimental work. The interaction not with the process itself, but with its model allows studying its situational behavior quickly and inexpensively. Based on the strength of the modern mathematical apparatus, computational experiments with object models allow studying and predicting the phenomenon in detail in all its aspects. Mathematical modeling algorithms are steadily improving, capturing increasingly new areas of knowledge. Most models are designed and implemented to predict short-term morbidity. That is driven by the needs of the anti-epidemics services for the timely preparation and implementation of efficient preventive, anti-epidemics, and therapeutic measures [13–16].

In the fight against COVID-19, contemporary technologies like deep learning, machine learning, and data science are helping. The healthcare system is greatly benefited by these strategies. Linear Regression, Support Vector Machine, Multi-Layer Perceptron, and Vector Auto-Regression are among the most popular methods among them. Machine learning can be a helpful method for accurately assessing, screening, following, forecasting, and predicting the characteristics and trends of the COVID-19, according to Rahman et al. [17].

Deep learning has created a new pathway in the healthcare system. The healthcare system has made considerable strides toward autonomous disease identification with the use of deep neural networks, including tumor detection, cancer cell detection, chest disease detection, and genomic sequence analysis. A combined architecture of Convolutional Neural Network consisting of 20 layers, On the basis of the automatic feature extraction from X-ray pictures, the COVID-19 identification using Recurrent Neural Networks and Long Short-term Memory is significantly impacted [18–20].

## Methods

When setting the research problem, the team of authors was guided by the following considerations. Since viral infections like COVID-19 are characterized by an exponential increase in the number of cases at the initial stage of the epidemic, it would be very interesting to know the following: is it possible to make some forecast of the development of the epidemic from the first data on the number of cases? Since the answer to such an important question is far from obvious, the authors first wanted to answer the following questions:

1. Is it possible, based on a limited number of cases, to draw conclusions about how the epidemic spread of a particular infectious disease corresponds to its viral nature?
2. Is it possible to make a more or less accurate prediction based on the information already available about the behavior of the disease?

To answer these questions, the authors used a feature that is very characteristic of a viral disease, namely, the exponential spread of the disease in the initial period of the epidemic. Since the growth rate must remain constant in the exponential nature of the epidemic, the authors therefore needed to make sure that this rate is stable. If its stability is not confirmed, then the expected prediction results are unlikely to be relevant since then the dynamics of disease growth will differ from the typical behavior of viral infections.

With an affirmative answer to these key questions, one can proceed to forecasting. To predict for a long period, it is necessary to have information about the nature of the disease. A simpler task is to build a short-term forecast for the very near future when there is no doubt that the behavior of the infectious agent will not change much. In this case, for forecasting, it would be optimal to use simple mathematical models for the development of infections, for example, the SIR model.

**Fig. 1:**
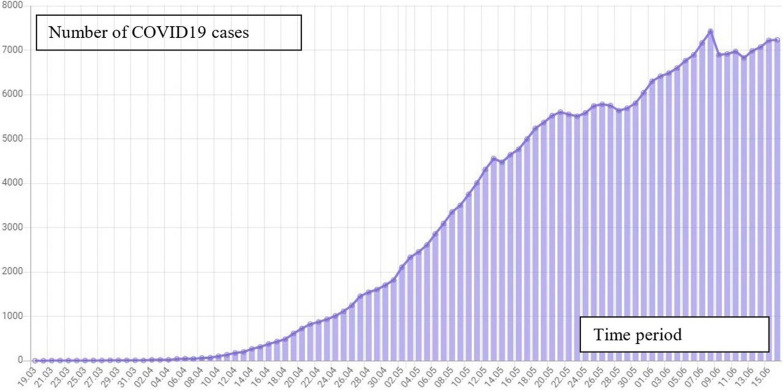
Dynamics of COVID-19 prevalence in Nizhny Novgorod region

The study was carried out using the data of Nizhny Novgorod region with 11,499 confirmed COVID-19 cases by early June 2020. Based on monitoring data as of June 15, 2020, the number of people infected with the virus increased by 330, and the number of people who died was 125. The number of patients who recovered and were discharged reached 4612 (Fig. 1).

The massive outbreak of COVID-19 pathogen in Nizhny Novgorod region has posed many challenges to the management of the region and regional healthcare. It required challenging management decisions, the important basis of which may be the information obtained by the timely use of prognostic algorithms for the spread of infection.

**Fig. 2:**
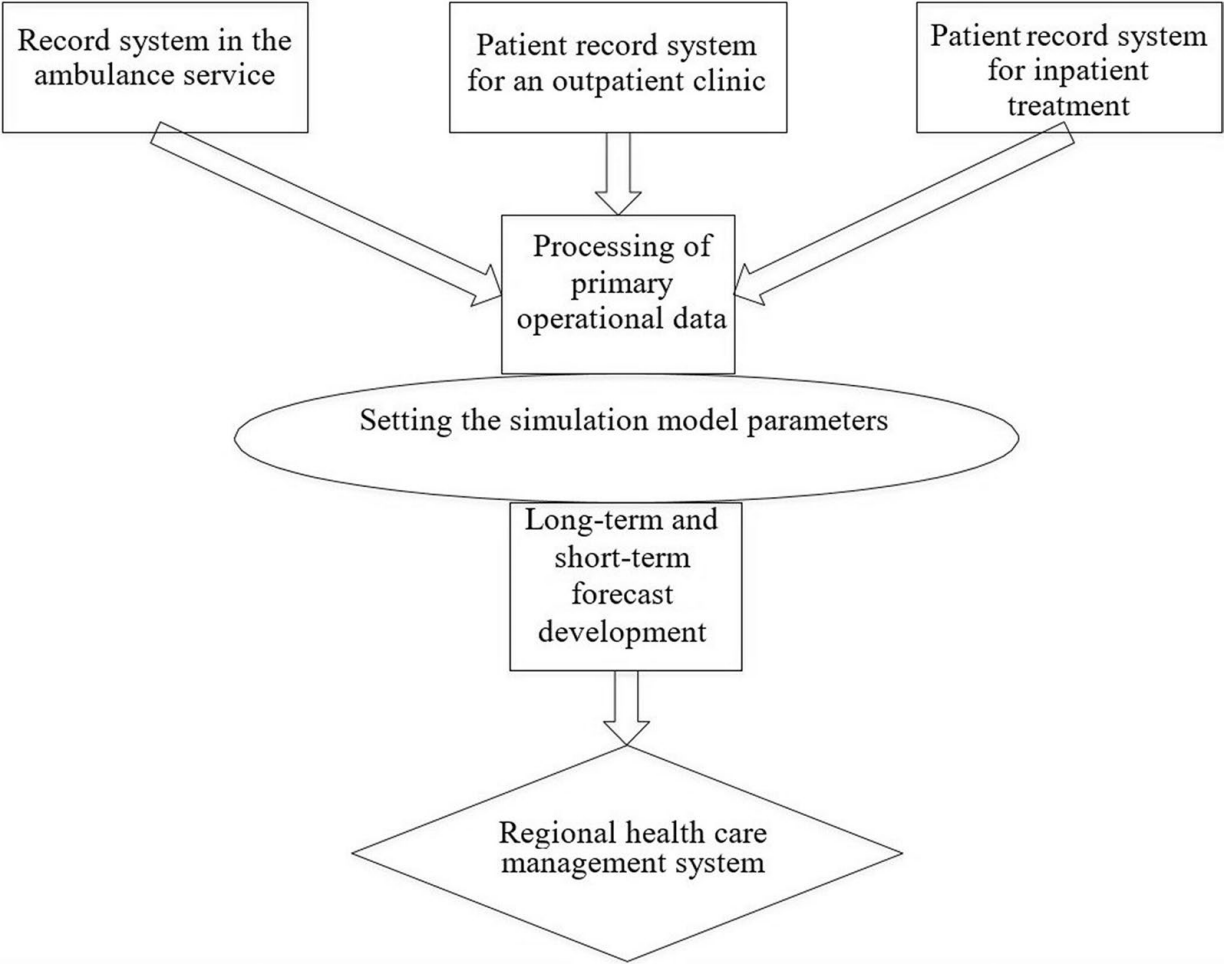
Flow chart of VDD Data Analysis System

The prediction of values for virus disease propagation dynamics (VDD) parameters is carried out using a data analysis system built according to the following scheme (Fig. 2).

The purpose of using a VDD online data analysis system is not to design a template that considers as many factors as possible, but rather to obtain satisfactory projections of disease dynamics with minimal time and resources. There are a number of prognostic algorithms to analyze the perspectives of viral infections, ranging from simple to very complex ones. Hence, the major problem in developing a prognostic model is to choose the best option. In the COVID-19 pandemic, which is not only threatening, but is developing rapidly, simple prognostic algorithms describing possible scenarios are the most appealing. Such algorithms are particularly convenient for fast-evolving situations.

According to scientists from the Singapore's University of Technology and Design, the COVID-19 outbreak in Russia was expected to end in early autumn 2020. As it is evident today, that was a miscalculation. Unfortunately, the computational algorithm has not been released to the public. All what is known is that the SIR model has been used, which was once considered the gold standard for describing the spread of infectious diseases: Susceptible, Infected, Recovered. It was introduced in the 1920's and has an extensive history of application [21]. According to the attitude towards the disease, the population is divided in groups: susceptible—S, infected—I, and recovered—R.

The rate of increase in the number of diseases is determined by the following formula:

$${\text{Xi}} = \left( {{\text{Xi}} + 1{-}{\text{Xi}}} \right)/{\text{Xi}}$$

**Fig. 3:**
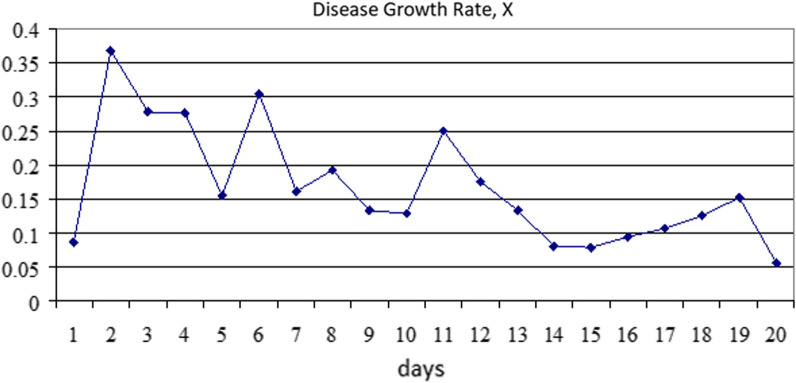
Disease Growth Rate

The results of the growth rate of the number of diseases are as follows (Fig. 3).

The growth factor X varies within a limited range, which confirms the hypothesis that the data in question can be considered data on a viral disease. Thus, the 1^st^ question can be answered in the affirmative.

To answer the 2nd question, it is necessary to assess the stability of the disease growth process. To do this, the authors construct the Shewhart (*Walter Andrew Shewhart*) map of this process. To do this, it is necessary to calculate the sliding range R using the formula:

$${\text{R}} = {\text{ABC}}\left( {{\text{Xi}} - {\text{Xi}} - 1} \right)$$

where ABC is the absolute value function, Xi, Xi − 1—current and previous value of the growth factor.

The calculation of the sliding range R is tabulated.

**Table:** 

X	R
0.0875	
0.367816	0.280316
0.277311	0.090505
0.276316	0.000995
0.154639	0.121677
0.303571	0.148932
0.160959	0.142613
0.19174	0.030782
0.133663	0.058077
0.128821	0.004842
0.249516	0.120695
0.174923	0.074594
0.13307	0.041853
0.080233	0.052837
0.078579	0.001653
0.093812	0.015233
0.107664	0.013852
0.12603	0.018365
0.151426	0.025397
0.056544	0.094883

To construct the boundaries of the Shewhart map, it is necessary to determine the average value of X (MX) and mR.

MX = 0.167; mR = 0.070.

The Shewhart map for average values should contain the upper and lower bounds, within which the values of the stable process should be contained. If the values obtained are outside the process boundaries, then this is an indication that the process is affected by special causes of variability. In the task of assessing the stability of disease growth rate, going beyond the boundaries of the Shewhart map will mean that there are no grounds to build a disease prognosis, since other extraneous factors act in addition to the virus impact factor.

The average moving range is multiplied by 2.66 to determine the Upper Natural Process Limit (UNPL), which is then added to the X-center map's line:

UNPL = M_X_ +(2.66*mR) = 0.167 + (2.66*0.070) = 0.354.

By dividing the average moving range by 2.66 and removing the result from the X-center map's line, one can get the lower natural process limit (LNPL):

LNPL =X−(2.66*mR) = 0.167−(2.66*0.070) = − 0.020.

**Fig. 4:**
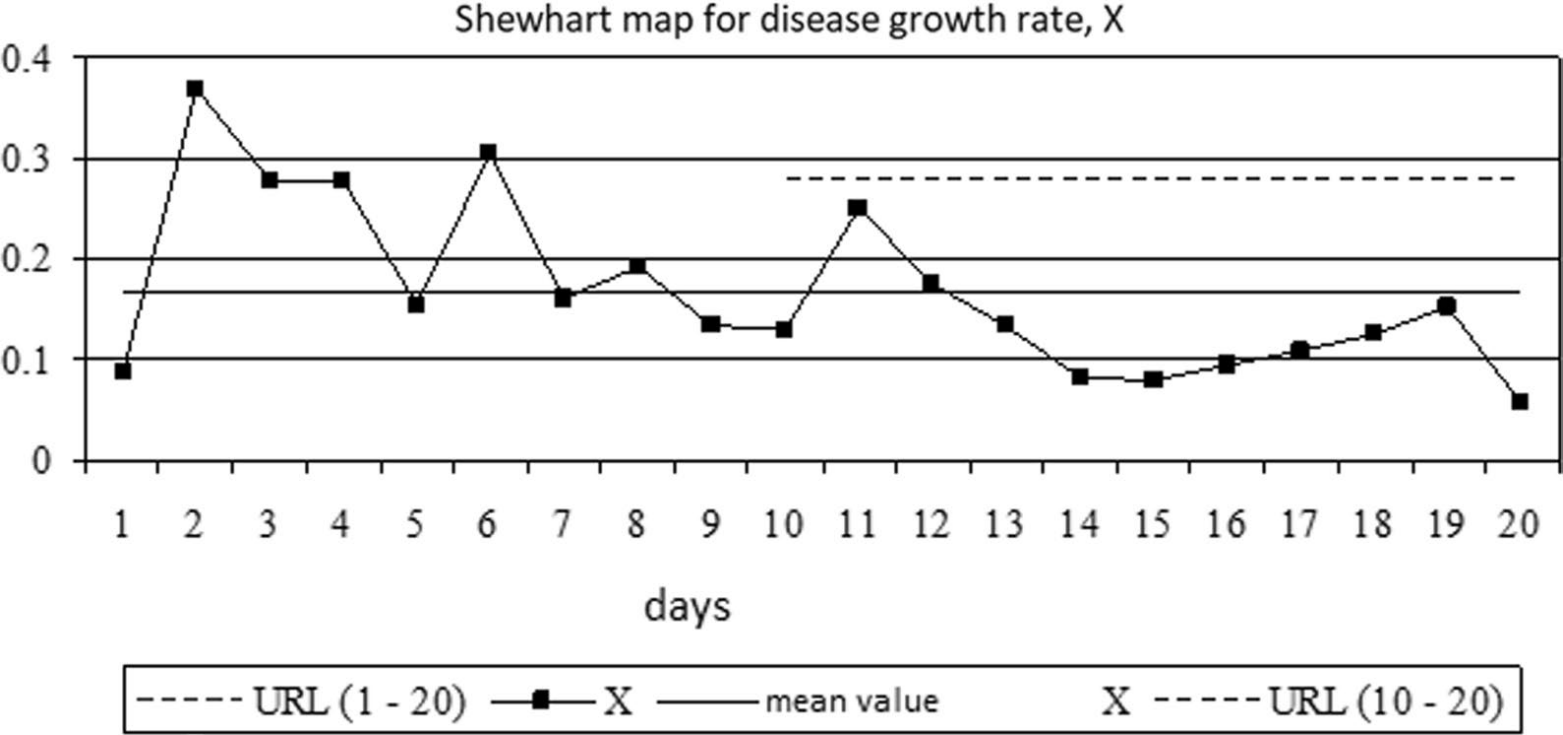
Shewhart map for disease growth rate, X

Since the lower natural limit of the process turned out to be less than 0, the authors will not take this limit into account. As a result, the Shewhart map for the growth rate of diseases will reflect the content of Fig. 4.

Analysis of the Shewhart map allows one to note the following features of the process:

- At the beginning of the process, the value of the coefficient of growth in the number of diseases goes beyond the upper natural limit of the process.
- In the second part of the process timeline, process stability increases.

Going beyond the UNPL border of the second point is explained by the small amount of information about the growth of the disease at the beginning of its spread. To test the assumption of a decrease in the variability of the disease growth rate, one needs to determine the UNPL for points from 10 to 20:

UNPL = MX +(2.66*mR) = 0.167 + (2.66*0.042) = 0.279.

The validity of the assumption is confirmed—the points of the process are within narrower boundaries.

One can draw the conclusion that it is possible to create an algorithm for predicting a disease after doing an analysis of a disease's growth rate stability.

In time, it is possible that S changes to I, and I to R. A simplified version of the SIR implies that:

1. The number of treatments per unit of time is proportional to the total number of infected individuals, i.e., each infected person has a fixed probability of recovery in units of time.
2. The number of infections is proportional to the product S × I. This hypothesis is based on the notion that the infection occurs through the so-called unsafe contacts, i.e., the contact between susceptible and infected individuals. If the total number of contacts among persons per unit of time is constant, and if the population is sufficiently mixed, the proportion of hazardous contacts should be commensurate with product S × I.

The mathematical model of SIR, describing the dynamics of changes in the number of potential patients (susceptible to a given disease), those who fell ill, and those who recovered (incl. died), is represented by a system of three equations:

1 $$\begin{aligned} \frac{dS}{{dt}} & = \frac{\beta IS}{N} \\ \frac{dI}{{dt}} & = \frac{\beta IS}{N} - \gamma I \\ \frac{dR}{{dt}} & = \gamma I \\ \end{aligned}$$

where N = S + I + R is the population size; S(t) is the number of susceptible individuals at time *t*; I(t) is the number of infected individuals at time *t*; R(t) is the number of individuals who have been infected at time *t*; β—intensity factor of contacts with subsequent infection; γ—intensity factor of infected individuals’ recovery (the value inverse of the average duration of the disease).

The first equation indicates that the variation in the number of healthy but susceptible individuals decreases over time in proportion to the number of contacts with infected individuals. After contact, the infection occurs, and the susceptible person becomes infected.

The second equation shows that an increase in the number of people infected occurs in proportion to the number of contacts between healthy and infected individuals and diminishes as they recover. The proportional coefficient β is one of the principal parameters of the mathematical model.

The third equation shows that the number of individuals recovered per unit of time is proportionate to the number of infected individuals. In other words, each person who has fallen ill must recover after a while. The coefficient of proportionality *γ* characterizes how a patient’s body adapts to a new virus [22].

As can be seen from the SIR model, the disease develops according to the ‘susceptible to become infected and then recover’ pattern. The following condition:

2 $$\frac{dS}{{dt}} + \frac{dI}{{dt}} + \frac{dR}{{dt}} = 0$$

Describes the invariability of the population and does not take into account deaths from the disease. The number of patients at a given time is determined by a parameter called the base reproduction number:

3 $$R_{0} = \frac{\beta }{\gamma }$$

It is generally accepted that the most important parameters in the SIR model are: characteristic time *t*—typical recovery time; reproduction rate R_0_—the ratio between infection and recovery rates. Parameter R_0_ can be considered the average number of people on whom an infected person spreads the virus over time before recovery [23–25]. The main feature of the SIR model is the epidemic transition: the VVD depends radically on whether the R is superior or inferior to one. At R_0_ < 1, the epidemic subsides; at R_0_ > 1, it develops, covering a large part of the population. The extent of coverage depends on the specific value of R_0_, which, in turn, depends upon the characteristics of the virus, the proportion of the vaccinated or recovered population, and measures taken to control the outbreak, i.e., various quarantine forms. For example, if $R_{0} = 2$, the number of those infected could represent approximately 80% of the total population.

Initial data for the first month of the pandemic (from 2020-04-08 to 2020-05-08) are shown in the table (Table 2).

**Table 2:** Baseline data on the development of the epidemic

No	I	R	No	I	R
1	67	13	16	942	60
2	74	13	17	1019	77
3	105	14	18	1122	92
4	138	14	19	1258	109
5	180	14	20	1465	109
6	204	20	21	1554	109
7	272	20	22	1617	177
8	318	21	23	1718	226
9	378	26	24	1834	289
10	432	26	25	2125	318
11	491	26	26	2349	346
12	620	26	27	2466	386
13	733	26	28	2628	398
14	832	28	29	2876	422
15	880	49	30	3117	493

Solving the prediction problem begins with determining the model parameters for the actual disease data. In the equation system of the model (1), the expressions for the base model parameters β and γ can be obtained (2):

4 $$\gamma = \frac{{\frac{dR}{{dt}}}}{I} \beta = \frac{{\left( {\frac{dI}{{dt}}} \right) \times N}}{I \times S} + \gamma$$

**Fig. 5:**
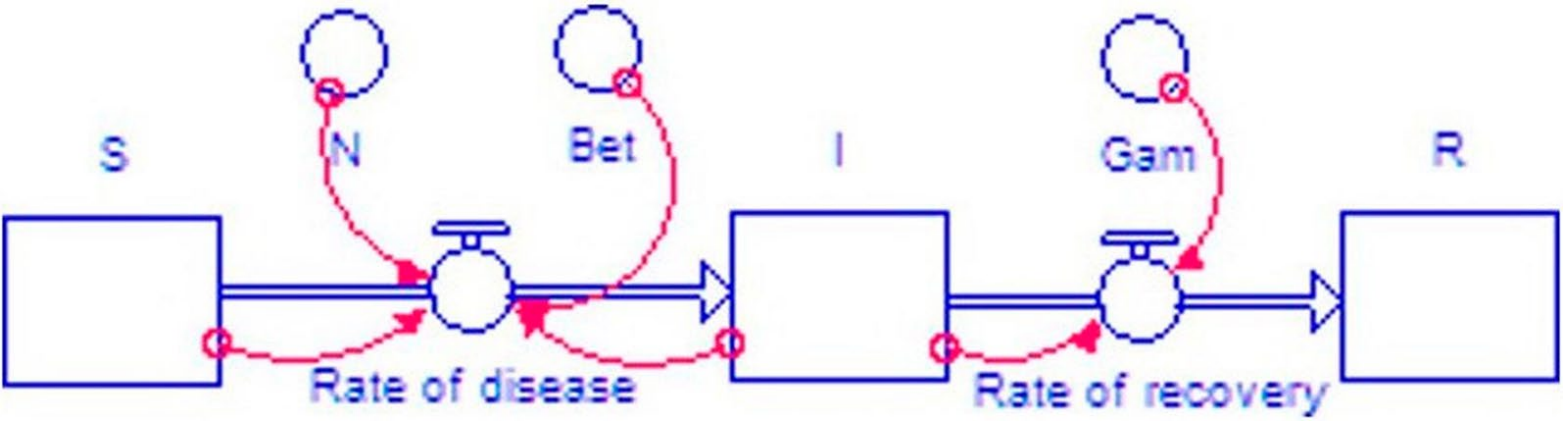
The average model of epidemic system dynamics in the form of flows and stocks

Endpoint dependence data from the SIR model were used in epidemic development calculations in the average model of epidemic system dynamics in the form of flows and stocks [17, 18] (Fig. 5).

**Fig. 6:**
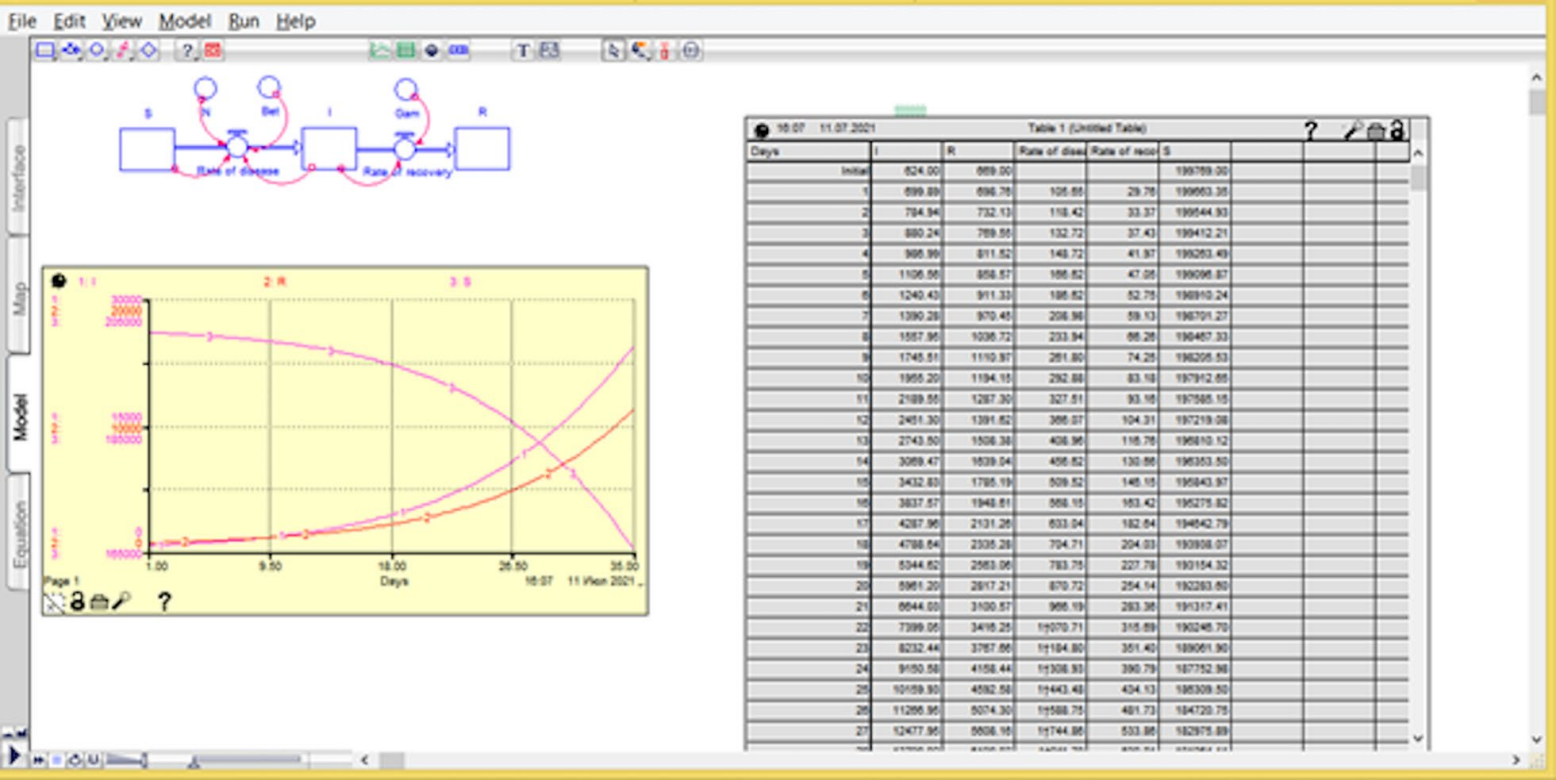
The computational window in iThink

Modeling and calculations were performed in iThink v9.0.2 software (www.iseesystems.com). This figure shows the computational window in iThink (Fig. 6).

Stock S allows one to enter data on the population, stock I characterizes the number of cases, stock R characterizes the number of recovered. The Rate of disease stream determines the number of cases per unit of time, and the Rate of recovery stream determines the number of recoveries per unit of time. The iThink program allows one to describe the relationships between model variables, as well as derive a system of equations for relationships between variables:

I(t) = I(t—dt) + (Rate_of_disease—Rate_of_recovery) * dtINIT I = 624.

INFLOWS:

Rate_of_disease = Bet*I*S/N.

OUTFLOWS:

Rate_of_recovery = I*Gam.

R(t) = R(t—dt) + (Rate_of_recovery) * dtINIT R = 669.

INFLOWS:

Rate_of_recovery = I*Gam.

S(t) = S(t—dt) + (- Rate_of_disease) * dtINIT S = 199,769.

OUTFLOWS:

Rate_of_disease = Bet*I*S/N.

Bet = 0.16.

Gam = 0.045.

N = 200,000.

**Fig. 7:**
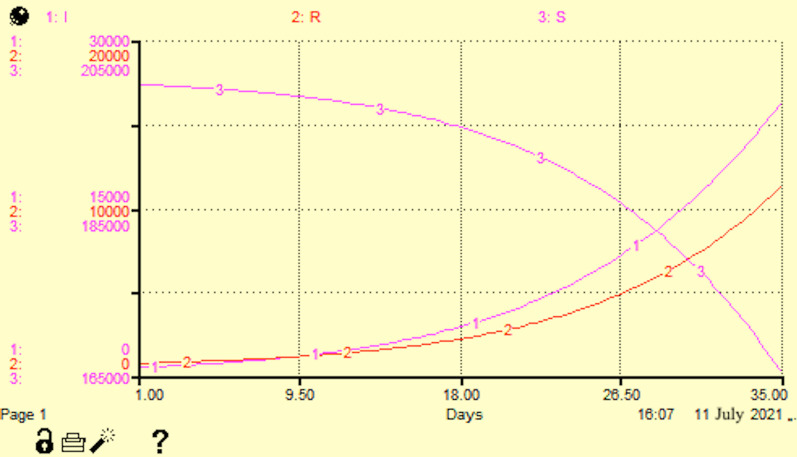
The results of calculations both in the form of a graph with curves and in tables

**Fig. 8:**
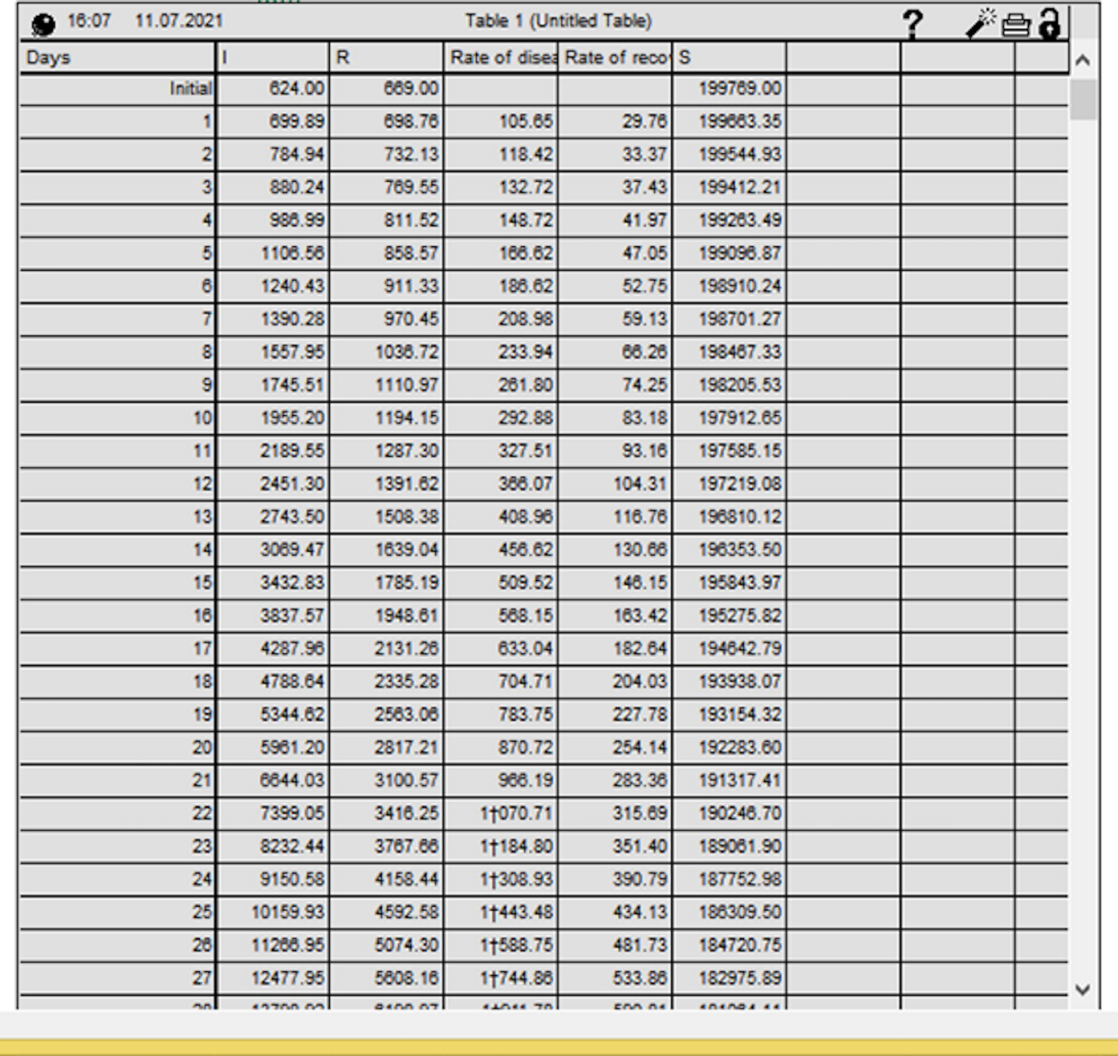
The results of calculations both in the form of a graph with curves and in tables

The iThing program allows one to display the results of calculations both in the form of a graph with curves and in tables (Figs. 7 and 8).

The calculation shall include the following initial conditions:

N = 300,000.

I = 48;

R = 8;

S = 299,944;

*β* = 0.2455e-0.0414t;

*γ* = 0.0041 + 0.0005t.

## Results

Actual data on the development of an epidemic are generally represented by the following datasets: the number of infected individuals detected, and the number of patients recovered. The number of infected cases (I) is defined as the difference between the number of cases established and recovered. The dR and dI increments were determined using a 3-point scheme that provided a more precise definition of derivatives. Parameters *γ* and *β* were determined by formulas (2). An example of calculation (the full table comprises 30 rows) of the parameters of model γ and β is given in Table 3:

**Table 3:** Calculation of model parameters

Date	Number of people recovered	Number of people infected	Incremental R	Incremental I	*γ*	*β*
	R	I	dR	dI	*γ* = dR/I	*β* = *γ* + dI/I
08.04.2020	13	67				
09.04.2020	13	74	0.5	19	0.0074	0.2910
10.04.2020	14	105	0.5	32	0.0067	0.4391
11.04.2020	14	138	0	37.5	0	0.3571
12.04.2020	14	180	3	33	0.0217	0.26087
4.05.2020	386	2466	26	139.5	0.0110	0.0704
5.05.2020	398	2628	18	205	0.0072	0.0904
6.05.2020	422	2876	47.5	244.5	0.0180	0.1111
7.05.2020	493	3117	44	250	0.0152	0.1022

**Fig. 9:**
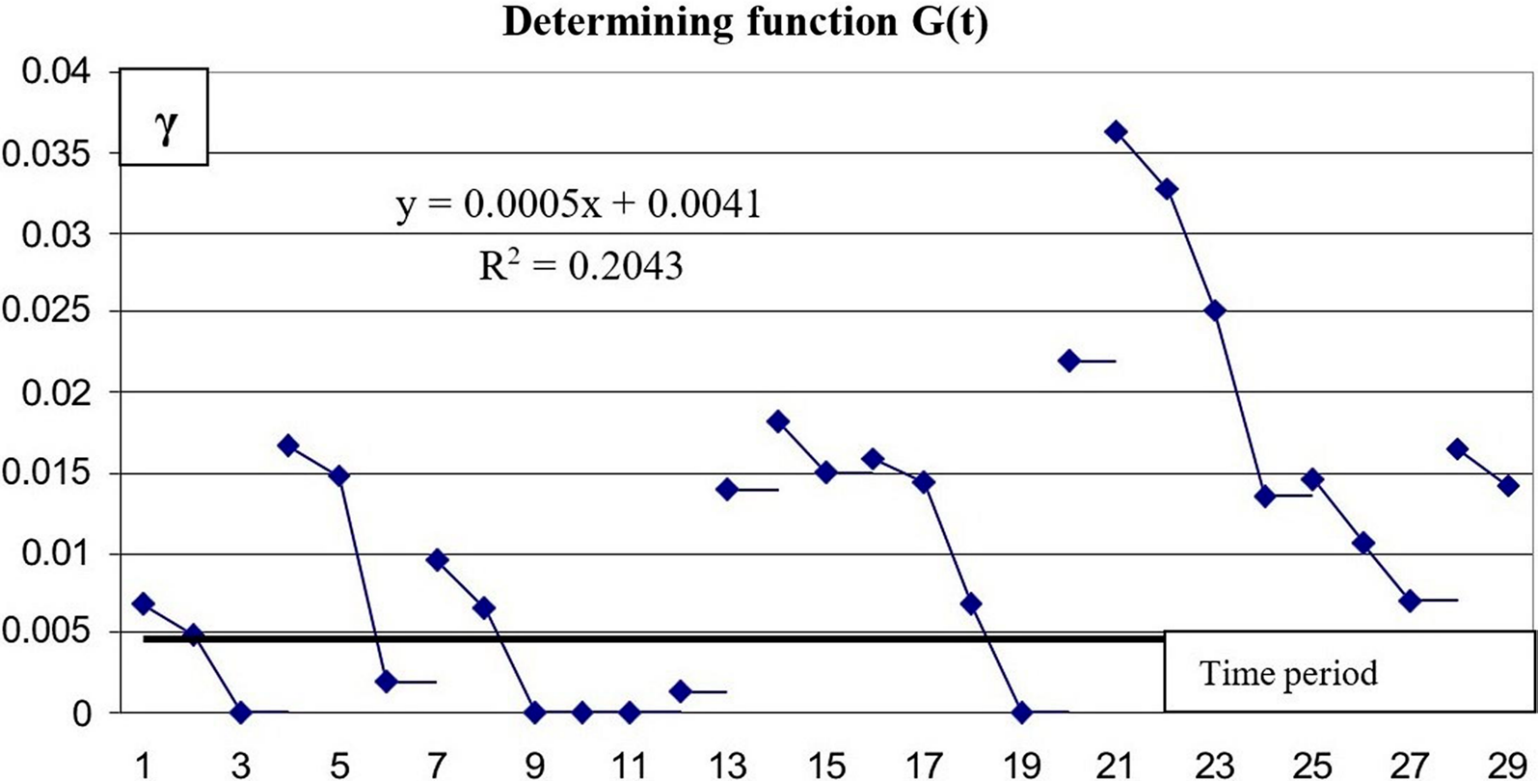
Changes of parameter γ with time (G(t))

**Fig. 10:**
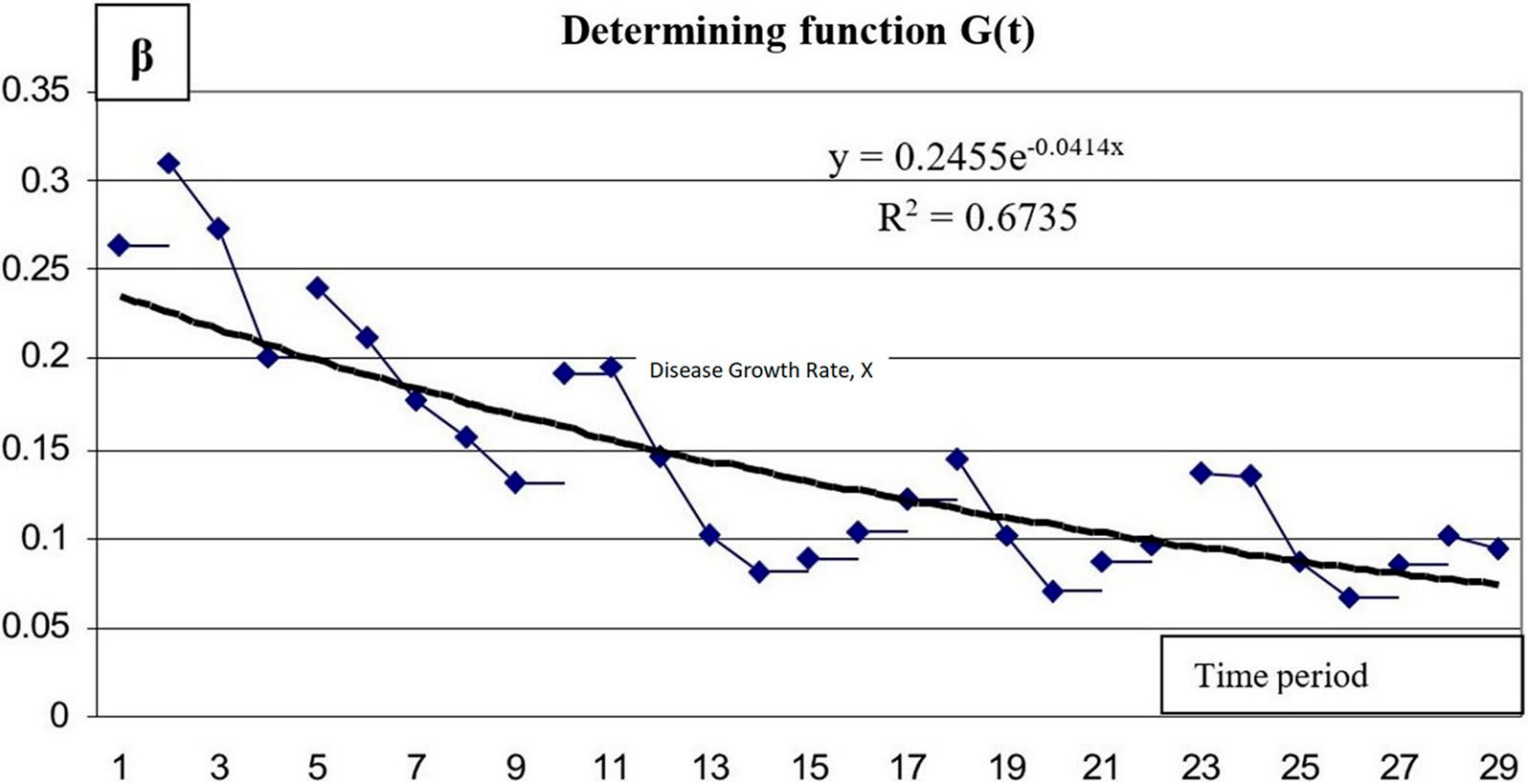
Changes of the parameter *β* with time (B(t))

Changes in *γ* and *β* parameters during the outbreak show that these parameters are not constant in the real epidemiological situation, and they present both random differences and a trend line (Figs. 9 and 10):

There is a large amount of computing data associated with a small number of individuals who recovered during the first month of the disease. It allows using only a linear function to estimate the calculated data on the number of people who recovered from the disease.

5 $$\gamma = 0.0041 + 0.0005 \times t$$

The high variability of the data defined the low coefficient of determination R^2^ = 0.2.

Data on variation in the intensity of *β* (B) infection allow choosing an exponential dependence as a proxy function.

6 $$\beta = 0.2455 \times e^{ - 0.0414 \times t}$$

A larger sequence of data on infection intensity *β* (B) results in a higher coefficient of determination, R^2^ = 0.6736.

**Fig. 11:**
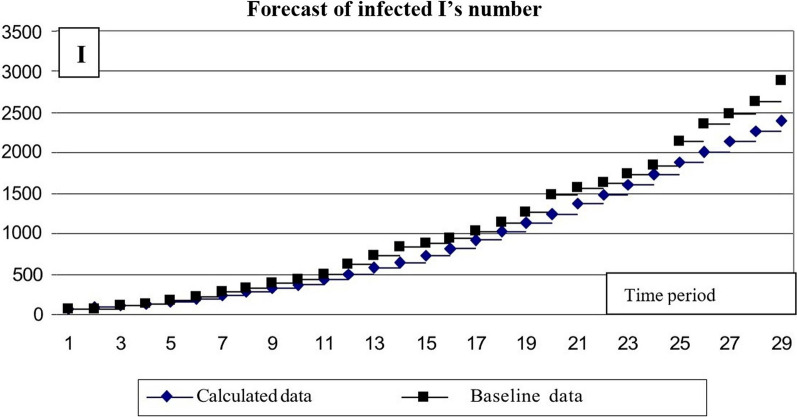
Calculation of the number of infected *I*’s

**Fig. 12:**
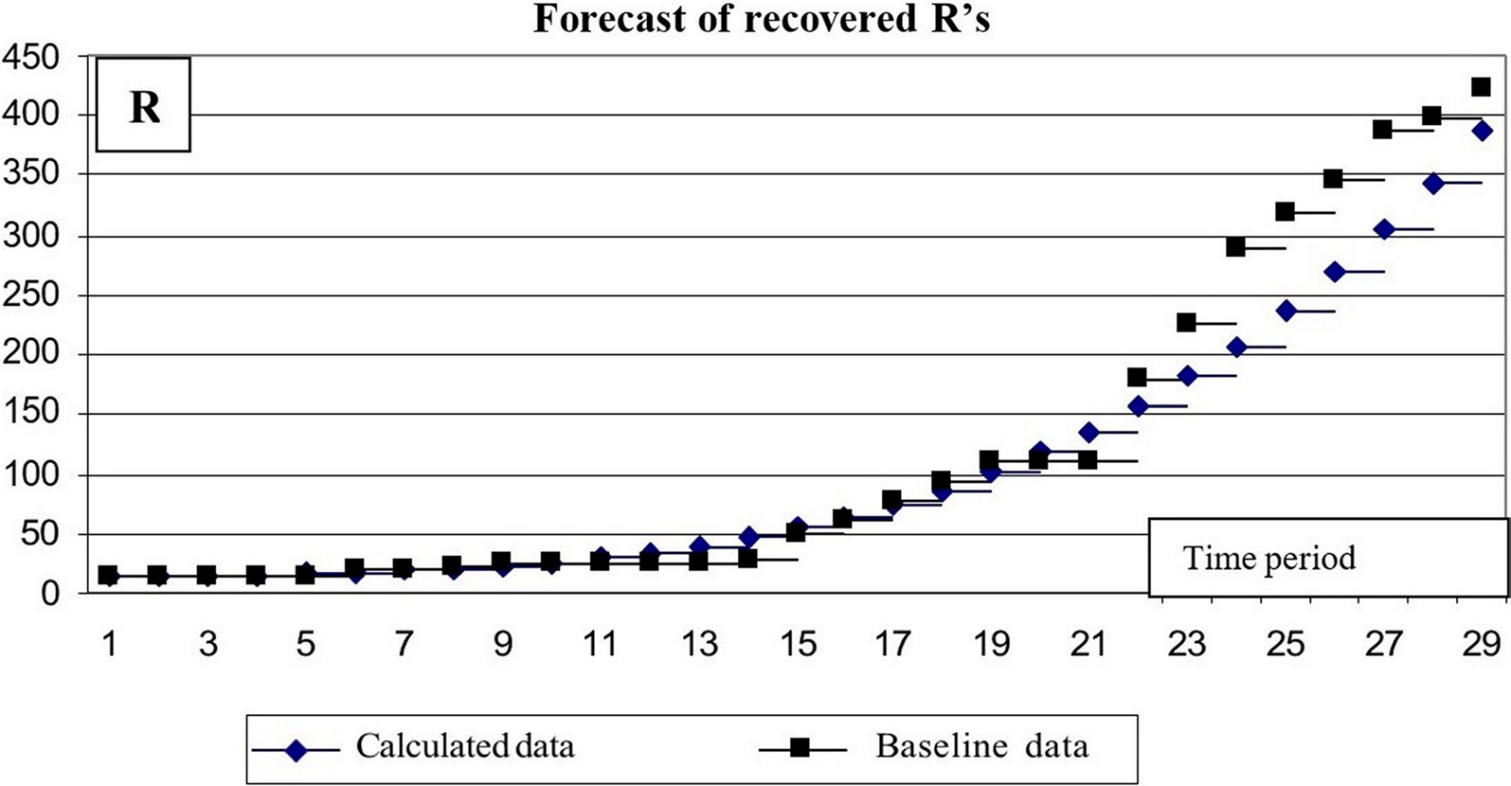
Results of calculating the number of *R*’s

The results of the calculations are presented as charts (Figs. 11 and 12).

**Fig. 13:**
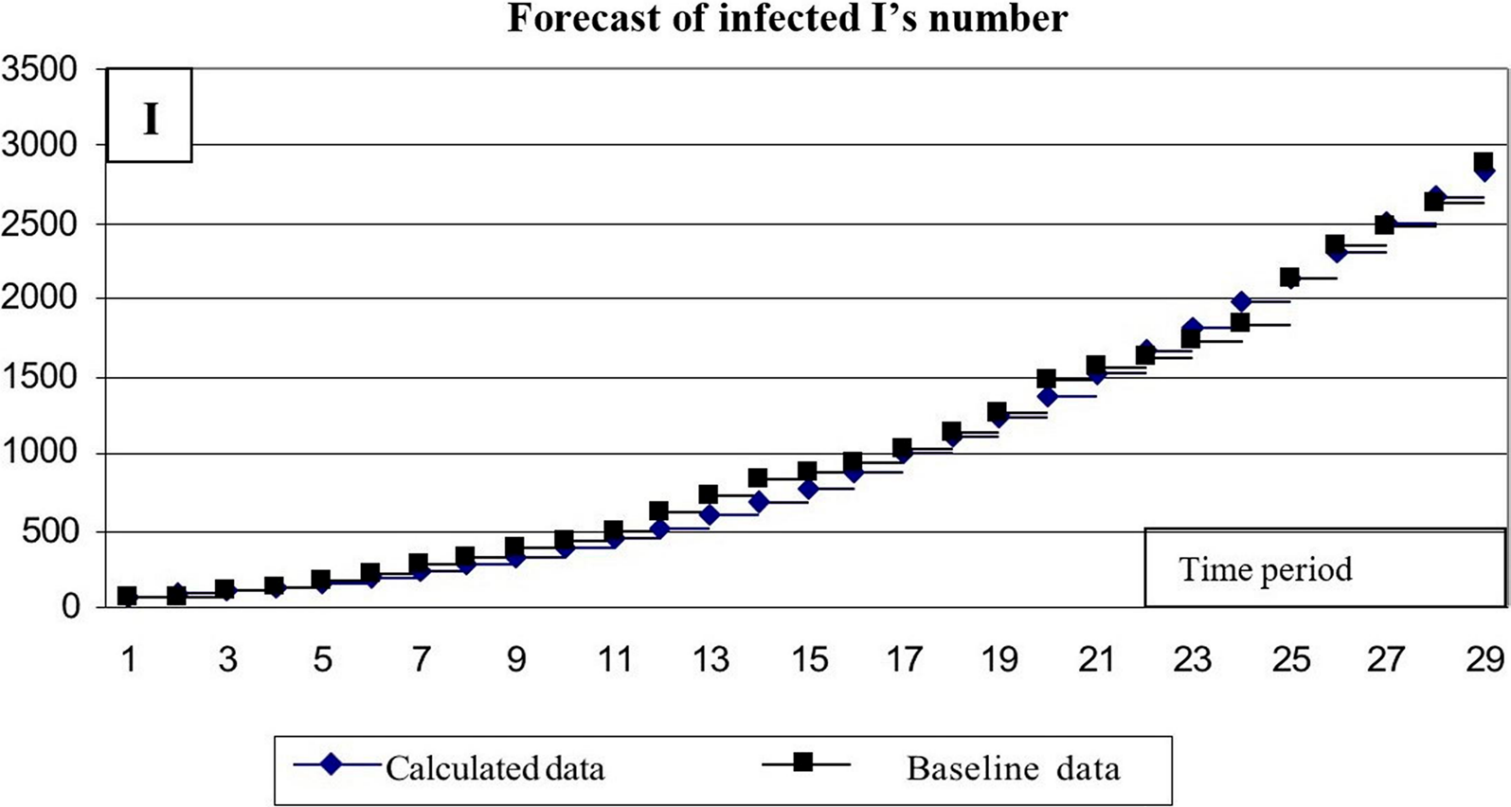
Results of calculating the number of I’s infected using the modified formula

The comparison of baseline and calculated number of infected *I*-s allows concluding that the discrepancy reaches a significant value at the end of the time range. To eliminate this discrepancy, approximate parameter β = 0.2455e-0.0414t can be adjusted. By changing this time-related parameter, a value can be found that provides a better match between the calculated and baseline data (Fig. 13).

The modified formula is as follows:

7 $$\beta = 0.2455 \times e^{ - 0.038 \times t}$$

In this case, the exponent index coefficient is reduced from 0.0141 to 0.039.

By changing one of the approximation formula parameters for the infection intensity, a fairly satisfactory agreement between the calculation data and the original data can be achieved. As a result, data from the first month of the outbreak can help determine the following parameters of the simulation model.

8 $$\begin{aligned} \beta & = 0.2455 \times e^{ - 0.038 \times t} \\ \gamma & = 0.0041 + 0.0005 \times \\ \end{aligned}$$

**t** is a model parameter that specifies a discrete calendar time since the start of the epidemic. At any given time point, individuals in the susceptible compartment may become infected by contacting individuals in the infectious compartment.

The equation for involves time-shifted terms and can be solved explicitly. The function **e** to the power of **x** is a special case of the exponential function, where the number **e**, otherwise called the Euler number, acts as the base. Otherwise, such a function is called exponential, it can be written in several forms: **=exp(x)**.

**Fig. 14:**
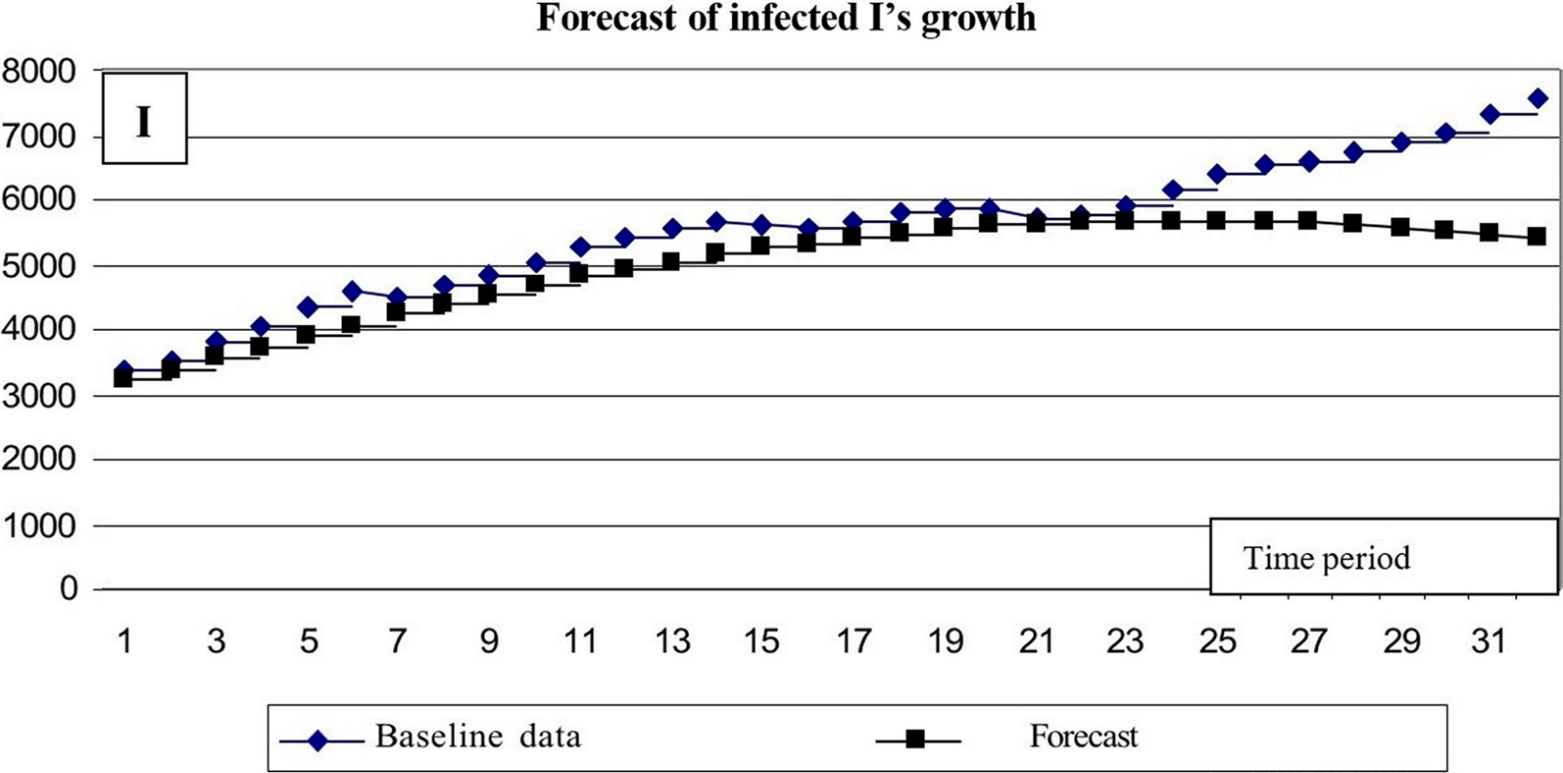
Results of predicting the number of *I*’s infected

β—intensity factor of contacts with subsequent infection; γ—intensity factor of infected individuals’ recovery. Based on these dependencies, the expected epidemic development for the following month, from May 8 to June 8, was calculated (Fig. 14).

The comparative results of the predicted values and data on the evolution of the epidemic in May–June allow concluding the correspondence between the predictive data and the actual situation.

To assess the forecast, the following criteria must be considered:

9 $$\sigma \left( X \right) = M\left( {\frac{{X_{ip} - X_{i} }}{{X_{i} }}} \right)^{2}$$

where σ is the mean square of the relative prediction error of the epidemic indicator; X^ip^—forecast value of the epidemic parameter on the forecast interval; X^i^ is the actual value of the epidemic parameter in the forecast interval.

The accuracy of the prediction for the number of infected *I*'s and the number of overexposed *R*'s was estimated (Table 4).

**Table 4:** Evaluation of the COVID-19 outbreak prediction accuracy

No	X_i_	X_ip_	σ_Ii_	X_i_	X_ip_	σ_Ri_
1	3376	3199.69	0.0027	510	550.04	0.0062
2	3531	3377.98	0.0019	649	615.34	0.0027
3	3781	3554.57	0.0036	661	685.89	0.0014
4	4039	3728.58	0.0059	694	761.82	0.0095
5	4343	3899.13	0.0104	744	843.26	0.0178
6	4594	4065.39	0.0132	764	930.29	0.0474
7	4511	4226.57	0.0040	1085	1022.97	0.0033
8	4683	4381.91	0.0041	1220	1121.34	0.0065
9	4809	4530.69	0.0033	1323	1225.42	0.0054
10	5040	4672.26	0.0053	1373	1335.18	0.0008
11	5282	4806.03	0.0081	1410	1450.6	0.0008
12	5413	4931.47	0.0079	1539	1571.61	0.0004
13	5572	5048.09	0.0088	1637	1698.13	0.0014
14	5657	5155.49	0.0079	1756	1830.03	0.0018
15	5609	5253.32	0.0040	2006	1967.2	0.0004
16	5569	5341.33	0.0017	2257	2109.46	0.0043
17	5649	5419.28	0.0017	2398	2256.63	0.0035
18	5806	5487.04	0.0030	2470	2408.53	0.0006
19	5852	5544.52	0.0028	2652	2564.93	0.0011
20	5830	5591.7	0.0017	2905	2725.6	0.0038
21	5719	5628.61	0.0002	3244	2890.28	0.0119
22	5778	5655.34	0.0005	3466	3058.72	0.0138
23	5896	5672.02	0.0014	3637	3230.64	0.0125
24	6141	5678.84	0.0057	3693	3405.75	0.0061
25	6402	5676.03	0.0129	3747	3583.76	0.0019
26	6523	5663.86	0.0173	3980	3764.38	0.0029
27	6598	5642.62	0.0210	4252	3947.28	0.0051
28	6718	5612.66	0.0271	4451	4132.17	0.0051
29	6887	5574.33	0.0363	4612	4318.74	0.0040
30	7025	5528.03	0.0454	4795	4506.66	0.0036
31	7300	5474.16	0.0626	4837	4695.64	0.0009
32	7564	5413.14	0.0809	4878	4885.37	0.0000
		σ(I)	0.0129		σ(R)	0.0058

**Fig. 15:**
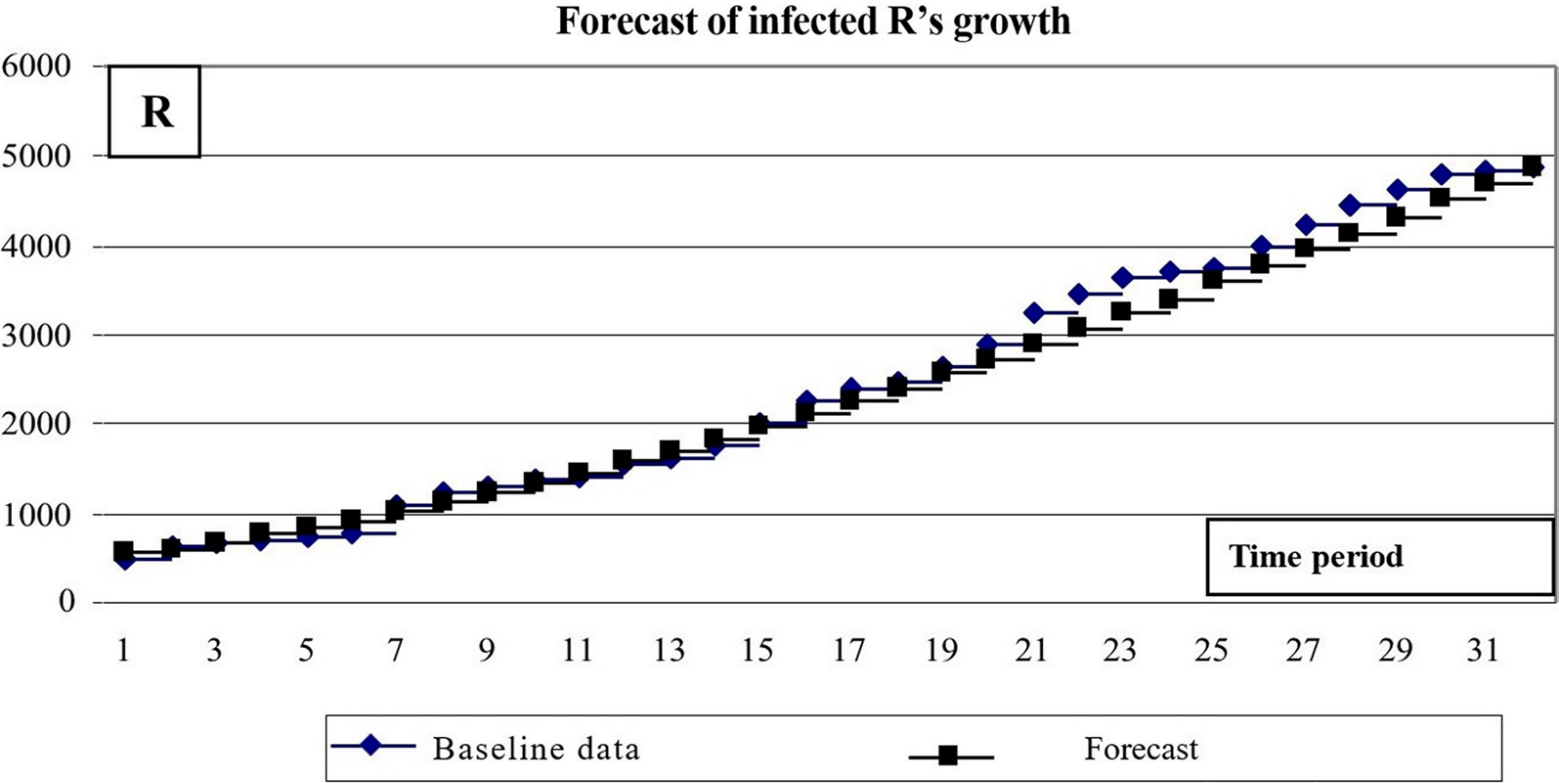
Results of predicting the number of people who have had a disease

The calculations yielded the following results (Fig. 15):

- Mean square of relative error for number of σ(I) infected = 0.0129,
- The mean square of the relative error of the number of people who have had a disease σ(R) = 0.0058.

## Discussion

To date, an entire clone of succeeding SIR models using more complex prognostic algorithms has been known. That is because at different times and under different circumstances, scientists have had to consider a variety of additional epidemic factors [26–28]:

- SIRS—susceptible-infected-recovered-susceptible. Suitable to describe the propagation dynamics of infections following which only temporary immunity occurs.
- SEIR—susceptible-exposed-infected-recovered. It considers the particularities of the spread of infections during a pronounced incubation period.
- SIS—susceptible-infected-susceptible. Suitable for describing the spread of infections without developing immunity.
- MSEIR—maternal derived immunity-susceptible-exposed-infected-recovered. The model considers children whose immunity is acquired in utero.

Although the mathematical models mentioned are more accurate, they do not take into account: the scope of anti-epidemics/quarantine measures, such as strict observance of social distance and masking regime; climatic conditions; the age of the population; logistics of passenger flows between countries and regions; immunity features, except for MSEIR; and other factors that can significantly affect the accuracy of forecasting. However, major drawback is the complexity of their use, which makes them much less convenient for forecasting and assessing the situation in extreme situations, when decisions need to be made in a situation where time, resources, and reliable information are not sufficient [29–31]. This may be supported by an example using the SEIR pattern, whose algorithms were used to make decisions regarding the introduction of restrictive measures in the city of Chicago. Unfortunately, it could not make a significant contribution to stopping the spread of the epidemic in the United States [32].

Mathematical modeling of the COVID-19 pandemic following the SEIR variant was also performed by Celestial experts, considering the peculiarities of Chinese population migration, the role of public health, and the variability of the incubation period. It served as the basis for introducing a system of restrictive measures by the country's government, which helped to combat the pandemic. However, any human-caused suppression of natural epidemic evolution is an interference with the free development of events that changes the conditions for the formation of herd immunity, which may even make the population more vulnerable to repeated epidemic waves [33, 34].

Prediction of the outbreak peak in the city of Moscow (with an error of 2 days and 174 less cases detected than in reality) was obtained by a group of Russian scientists from March to May 2020 using amendments from multiple authors of the SEIR model under the acronyms SEIR-D and SEIR-HCD. They proposed a prognostic scenario for disease progression in the city of Moscow and the Novosibirsk region for different numbers of data tested. At the same time, they believe that the use of coarser mathematical models that take into account fewer homogenous groups is justified only in the case of the availability of larger statistical datasets and a shorter time horizon. However, for modelling and prediction, additional constraints on model parameters other than the mortality parameter were not taken into account. Furthermore, they emphasized that there was no need to generate cluster calculations [35].

It is common knowledge that the risk of infection is by no means constant over time. Control strategies can alter the frequency of human interaction, which is considered to remain constant in simpler models, as a pandemic progresses. The frequency of interaction will change as a result of countermeasures like masks, social isolation, and social distancing, slowing the spread of the pandemic. This is connected with the manifestation of the phenomenon of nonlinear parametric resonance in dynamic systems. If the value of the integral in the mathematical expression

$$\frac{1}{T}\int_{0}^{T} {\frac{\beta \left( t \right)}{{\mu + \gamma }}} dt < 1 \Rightarrow \mathop {\lim }\limits_{t \to + \infty } \left( {S\left( t \right),I\left( t \right)} \right) = DFE = \left( {N,0} \right)$$

is greater than 1, then this means that the disease will not disappear, and there may be such resonances. One can discover, for instance, that the output is a periodic function whose period is a multiple of the input period when the changing contact frequency is used as the system input. This enables the relationship between the period of fluctuations in the frequency of contact and the pseudo-period of damped oscillations near the endemic equilibrium to be used to explain periodic epidemic breakouts of infectious diseases. It is noteworthy that the behavior of infectious disease waves can occasionally be chaotic or quasi-periodic [33]. A model for estimating the likelihood of global spread of a pandemic was recently developed by Valdez et al. [36]. How a stable equilibrium is eventually established is a crucial question for any dynamical system. Do the trajectories change as they get closer to the equilibrium state or do they usually go from one to the other smoothly? The SIR model has high oscillatory dynamics, but as the system balances over time, the magnitude of these fluctuations reduces.

An attractive aspect of the SIR model is that it can be easily modified to simulate vaccination. For this, a special additional parameter V for vaccinated persons is usually used. Using this approach provides an accurate infection-free periodic solution for a fluctuating epidemic situation, which is very attractive even on a global scale if the vaccination rate is high enough. Moreover, this approach demonstrates that if the level of vaccination is less than some critical value, then the disease continues to persist [37].

The SIR model extension can be used to describe the effects of lockdowns within a population, in particular, to simulate the decay of an epidemic over time by reproducing a lattice network model of the spread of an epidemic based on concepts taken from percolation theory. Percolation theory in statistical physics and mathematics defines how a network behaves when new nodes or links are added. To accurately determine how far a virus has spread throughout a region or the entire country and to put appropriate lockdowns in place in each specific location, tracking sick people in a population and their movements is crucial [38].

It is noteworthy that the SIR model is the base one. In the classical version, it does not provide for the possibility of vaccinating the population; herd immunity is assumed to be acquired naturally. Very often, the standard SIR model seems too simple and unrealistic, as it does not take into account the life cycles of the population and assumes that human is contagious immediately after infection. However, for example, in the SEIR model, it is assumed that the infection has a latent period, during which individuals are infected, but not yet infectious. The SEIR model grows more slowly after a pathogen invasion, despite the fact that the SIR model and SEIR behave similarly at equilibrium (when the parameters are scaled appropriately). This is because individuals must first pass through the exposed class before they can contribute to the transfer process. In addition, one of the serious disadvantages of SIR is that people who died from this disease are among the recovered [39]. However, the basic SIR model serves as a starting point for developing more complex models that include characteristics such as demographic groups with different health risks, the impact of public health interventions, natural birth and death rates, and the impact of stochasticity. New pathogens are constantly emerging in the world, which can lead to a pandemic and which humanity has yet to defeat. Therefore, science does not stand still, offering more and more new methods for predicting the behavior of infections, here the role of mathematical modeling is great, which is based on SIR. The topic considered in the article is very extensive and relevant, especially in the context of our time, when humanity has been suffering from a coronavirus infection for several years now.

Thus, the findings of this study confirmed the effectiveness of using simpler SIR models for operational forecasting. Their low precision can be compensated by an adaptive parameter calibration based on monitoring of current situation and real-time data updates. It should be noted that the results of forecasting the number of outbreaks are entirely consistent with data on the evolution of the outbreak. Available data volume was used in the following way: in the 1st month of the epidemic to build a forecast model, and in the 2nd month – to compare the forecast results with actual indicators. The equations of changes in the parameters of γ and β models were calculated based on the data of the first month.

As COVID-19 is a new virus, the R_0_ parameter is not known exactly. The R_0_ during the outbreak at Wuhan City was estimated by different authors between 2.2 and 6.5. The problem with the coronavirus is that it has a long incubation period of up to 14 days, and probably a very large proportion of carriers remain asymptomatic. These asymptomatic carriers play an active role in spreading infections. Different R_0_ values may be present even in the same city, but in different segments of society. Consequently, the issue of determining R_0_ for coronavirus is not completely closed. At R_0_ < 1, the pandemic subsides, while at R_0_ > 1, it spreads. According to data obtained in this study, the coefficient R_0_ changed exponentially, decreasing from R_0_ = 5.0 to values below 1 with time.

In the present work, the authors could not pass over in silence the latest achievements in the field of machine learning architecture of neural networks, which could be used to support and make the proposed model more efficient. It is no secret that an already trained and debugged neural network does not respond well to the transition from task to task. Therefore, for each especially specific task, this neural network must be retrained. Significant progress in solving the problem of universality has been made at Google Brain. The neural network, designed to work simultaneously on sets of tasks from different domains, had a complex architecture with blocks for processing different input data and generating a result [40]. Expert level users sometimes need information about data markup. These labels are stored in the database and are available for use by the orchestrator with datasets for training, validation, or testing. The question of what data should be marked up can be defined manually or programmed into the orchestrator. For this, input production data are used when the neural network worked correctly, but uncertainly; and when it was confident, it did not work right. This is one of the foundations of active machine learning architecture.

In implementing a full-scale inclusive health policy, the state provided the Nizhny Novgorod region with all the necessary organizational, material, and financial support. The Government of the Nizhny Novgorod Region, acting in full compliance with the recommendations of the Government of the Russian Federation, has taken measures to prevent the spread of the COVID-19 both to neighboring regions and the penetration of coronavirus from them. These measures included: severe restrictions on the movement of migration flows through total checks with the identification of infected and disinfection of vehicles, the transition of educational institutions to an online learning format, the closure of public catering establishments, shopping centers, etc., the maximum transition of workers to teleworking. There was also cancellation of public events, limiting the number of people who can gather, decrease in public transport, the obligation to stay at home (especially for children and people of retirement age), quarantine measures in health facilities, as well as travel restrictions not only inside country, but also abroad. The measures described have been very effective in reversing the upward trend in the number of cases and deaths from the COVID-19.

The features of the state health policy during the pandemic were as follows: a self-isolation regime was introduced in the Nizhny Novgorod region, which led to an almost complete obstruction of the movement of the population between neighboring regions. Therefore, in fact, it was decided not to include other regions when considering the application of the simulation model. This could certainly make it excessively heavy and not in the best way affect the accuracy of forecasting. In addition, turning again to the policy of the constituent entity of the Russian Federation in the field of healthcare, it should be noted that during the pandemic, for objective reasons, many medical institutions had to temporarily abandon some non-urgent planned surgical interventions and procedures, since a large number of medical personnel were sent to work in COVID-19 hospitals. However, this did not lead to any negative consequences associated with an increase in mortality or social tension in society. Similar indirect effects of the COVID-19 epidemic in other countries have often been more severe in this respect. Poor citizens and disadvantaged groups were more likely to miss out on basic health care there. However, in the Nizhny Novgorod region and in Russia as a whole, such unfavorable phenomena were avoided thanks, among other things, to the use of the model proposed here.

As the incidence of COVID-19 declined, the provision of medical services, which were temporarily suspended, was promptly resumed. Decisions on changes in the provision of services by the leadership of the Ministry of Health of the Nizhny Novgorod Region were made based on accurate and timely data on the real demand for the main set of essential medical services. The COVID-19 pandemic has exposed dangerous gaps in health system preparedness, coverage, and access to health services. Simulation models must play an objectively important role in the prevention of such risks.

## Conclusions

In the event of an epidemic, forecasting is necessary to determine how many hospital beds, ventilators, and how many months' worth of supplies of medical personnel protective equipment should be made. It is up to officials to determine if the current limitations and prohibitions are adequate or if more need to be put in place. To maximize productive efforts, judgments that turned out to be useless should be canceled so that the model accurately captures reality and may be used to determine the effectiveness of each newly adopted measure. With the help of both the SIR model and its other modifications, a lot can be explained and taken into account, but they cannot absolutely accurately predict something in the distant future. It is a matter of finding the sweet spot where the system is easy enough to learn to be accessible. Alexander Pope wrote about this: “There is a certain majesty in simplicity which is far above all the quaintness of wit.”

The algorithm described in the paper provides a real quantitative basis for prediction, which is required to understand the VDD and the impact of surveillance activities on it. At the same time, it is important to note that the key information for decision-makers in the region should be real-time follow-up data on the epidemiological situation. Using the SIR model, a prognosis algorithm was proposed based on real-time COVID-19 data. The data analysis revealed an interesting feature: the *β* infection parameter is not a constant but decreases exponentially. However, a thorough examination of this item is beyond the scope of this paper. The reliability of the proposed algorithm is rather high, even though the results can naturally differ numerically over a long forecast period. Over a 10–15 day horizon, the forecast results normally coincide with the actual data. The authors believe that the model-based approach for disease prediction is much more valuable than the use of formal mathematical methods only since it enables functional inter-relationships between epidemiological variables to be taken into account.

Thus, in case of repeated pandemic waves of new coronavirus infection, the proposed algorithm can be effectively used as a convenient prognostic tool to timely adopt adequate measures and prevent the spread of COVID-19 both in a particular region and in the country in general. However, despite the great importance that predictive algorithms have, it should be clear that they can never be an integral substitute for objective reality. Their role is to serve as a useful tool for obtaining further information, which will certainly prove useful in controlling the spread of this dangerous infectious disease. To enhance the effectiveness of these measures, it is advisable to establish a constant and close professional interaction between epidemiological predictors and public health authorities. After emerging out of this crisis, it is of paramount importance to continue research problematic aspects of forecasting.

## Data Availability

The data will be available from the corresponding author (Andrey Reshetnikov) upon a reasonable request.
